# Family and Educational Strategies for Cyberbullying Prevention: A Systematic Review

**DOI:** 10.3390/ijerph191610452

**Published:** 2022-08-22

**Authors:** Pamela Tozzo, Oriana Cuman, Eleonora Moratto, Luciana Caenazzo

**Affiliations:** Department of Cardiac, Thoracic, Vascular Sciences and Public Health, University of Padova, 35121 Padova, Italy

**Keywords:** cyberbullying, adolescents, prevention strategies, interventions

## Abstract

Cyberbullying can be described as a form of bullying carried out by an individual or a group through digital media with the intention to harm others. It has been recognized as a public health issue recently; however, of the vast literature published in recent years on the phenomenon, only a small part concerns strategies adopted to prevent and combat cyberbullying, and the effectiveness of these strategies appears to be scarce. We conducted a systematic review of the literature published in the last five years about different interventions studied to prevent and contrast cyberbullying. Our results show how most of the strategies currently developed focus on the educational aspect, involving schools and families. Other authors describe technology-based practices to set programs to reduce and prevent cyberbullying through the usage of digital instruments, the same used by minors themselves. Finally, remaining tactics use a more comprehensive approach, mixing tools already in use in the aforementioned strategies. Cyberbullying requires wide-ranging methods to combat it, involving the contribution of mental health professionals, educators, and digital experts cooperating synergically. Prevention and contrast instruments should be defined, implemented, tested, and combined in order to deal with cyberbullying.

## 1. Introduction

Although the Internet is characterized by many positive aspects for the personal and social development of young people (e.g., it permits to stay in contact with people all over the world and at any time), it has also accelerated the emergence of risky situations and digital forms of violence against them not only at the hands of adults but also through adolescents themselves [1,2].

This emergency is worsening because of the COVID-19 outbreak, which led governments to impose unprecedented stay-at-home orders to limit its transmission [3]. This has drastically reduced the rate of school attendance and ability for students to engage in proximity, resulting in traditional education methods’ replacement by the online learning format, conducted via digital services and devices, including e-mail, mobile phones, and social networking services (SNS). Noticeably, the more time children and adolescents spend on digital services, the more likely it is that they are exposed to digital violence, both nationally and internationally [4]. An example of this phenomenon is cyberbullying, which has emerged as a social and health concern, being recognized as a public health issue over the past few years [5]. Some contributions found in the literature have highlighted how an early intervention on the educational level is most appropriate [6,7].

At present, there is not a unified definition of cyberbullying in the world. Cyberbullying, in its broadest sense, can be defined as a form of bullying carried out by an individual or a group of perpetrators through electronic or digital media with the intention to harm others (it may include making fun of others, isolating, and spreading rumours about others) that involves repeatedly sending aggressive messages or other similar actions [8,9], or, according to the definition given by the Cyberbullying Research Centre, cyberbullying is “wilful and repeated harm inflicted through the use of computers, cell phones, and other electronic devices” [10].

In this sense, cyberbullying is a form of traditional school bullying for which ICT has provided a new platform. Recent studies have demonstrated that cyberbullying is positively connected with actual bullying: in other words, those who bullied others online were also more likely to bully others in real life and vice versa [11,12]. Considering that cyberbullying a new form of bullying, it too represents a dynamic of abuse of power exercised by a person, the bully, over another, the bullied, through aggressive and repeated behaviours that impact the victim’s private and social life [13]. However, there are other actors on this scene: supporters and bystanders, the first encouraging the perpetrator and the latter maintaining a neutral position [14].

As much as traditional bullying, cyberbullying can have long-term effects for its adolescent victims, with technology being one of the more important risk factors. Both cyberbullying victims and perpetrators are heavy Internet and mobile phone users and are more likely to engage in online risky behaviours than those not involved in cyberbullying with any role [15].

Within the recent past, many students have been aware that cyberbullying can have a terrible impact on the physical and mental health of teenagers, such as heartbreak, embarrassment, humiliation, and marginalization. Additionally, in a great number of cases, the cyberbullied develop anxiety, depression, and suicidal tendencies [16].

If we focus on the data regarding the prevalence of this problem, we can see that interest in this phenomenon has increased in recent years, with more and more data becoming available since the first study in the U.S. in 2000 [17]. Symbolic of this is that, for example, in Italy, specific rules for the prevention and the contrast to cyberbullying among adolescents were introduced only in 2017, with the law n. 71/2017, which does not introduce any type of crime related to the conducts of cyberbullies but launches some mechanisms useful to hinder it, avoiding the ignition of the criminal process. This law provides a legal definition of the phenomenon and indicates the role of schools in cyberbullying prevention, education, and rehabilitation through specific teachers’ training and the appointment of a referee among them in charge of coordination of prevention and contrast programs. This law introduces also a deadline of 24 h for an Internet website manager to take down a content after receiving a request for removal [18].

However, in face of the vast literature published in recent years on the phenomenon of cyberbullying, only a small part concerns the strategies adopted to prevent and combat this phenomenon, and the effectiveness of these strategies appears to be scarce.

This work constitutes a systematic review focused on illustrating what has been published in the literature in the last five years, from 2017 to 2022, in order to verify the effectiveness of the strategies adopted to combat the phenomenon of cyberbullying.

## 2. Materials and Methods

This review was performed in adherence with the Preferred Reporting Items for Systematic Reviews and Meta-Analyses (PRISMA) guidelines [19]. A systematic literature review regarding strategies or interventions adopted to address the cyberbullying phenomenon was conducted using a public electronic database (*PubMed*) using the following query strings separately: “cyberbullying adolescent survey”, “cyberbullying children survey”, “adolescent cyberbullying interventions”, “adolescent cyberbullying strategies”, “children cyberbullying interventions”, and “children cyberbullying strategies”, identifying a total of 1670 articles. The last search was performed on 31 March 2022.

Duplicates (n = 927) were then removed. Articles published before 2017 (n = 220) were excluded, and filters “English, Abstract, Full Text” were added, excluding other 10 papers and leaving a total of 513 articles to be screened.

Results management was performed with the use of Microsoft Office software such as Excel and Word. Zotero software was used to edit and organize the bibliography.

Two of the reviewers (O.C. and E.M.) carried out the initial search of the papers. In case of disagreements, the consensus of research supervisors (L.C. and P.T.) was required. Articles selected using the forementioned strings and filters were screened first through titles, then through abstracts, and lastly through a full paper’s reading. Reasons of exclusion were defined as non-relevance, intervention or strategy only descripted but not tested, and population sample consisting of adults.

A total of 405 articles were excluded after title reading and another 62 after abstract reading. In total, 46 articles were examined in full text for eligibility.

After full-text examination, 29 studies published between 2017 and 2022 were selected for qualitative synthesis.

**Figure 1:**
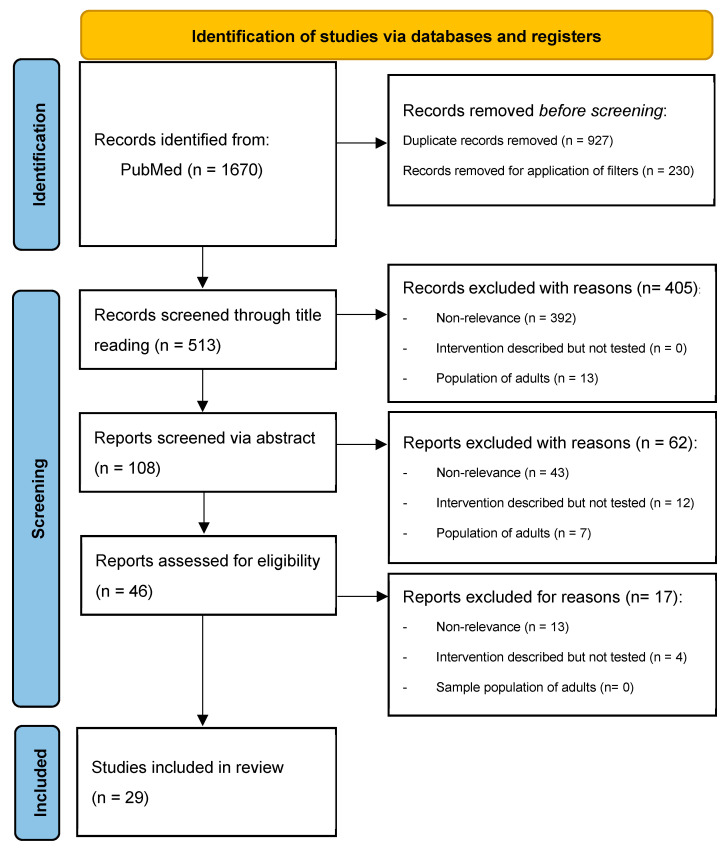
Preferred Reporting Items for Systemic Reviews and Meta-Analyses (PRISMA) 2020 flow diagram.

The number of articles included and excluded at each step are reported in the PRISMA flowchart (Figure 1).

## 3. Results

In order to make the results obtained in the current review more comprehensible to the reader, we organized the selected articles into four categories based on the type of intervention/strategy studied: (a) educational strategies involving school or families; (b) digital strategies; (c) hybrid strategies; and (d) other strategies.

### 3.1. Educational Strategies

We grouped in this category all strategies that involved a path of educational interventions at multiple levels. The settings in which these programs take place involve mainly schools and, in some cases, families or both.

#### 3.1.1. Educational Strategies Involving Schools

**Figure 2:**
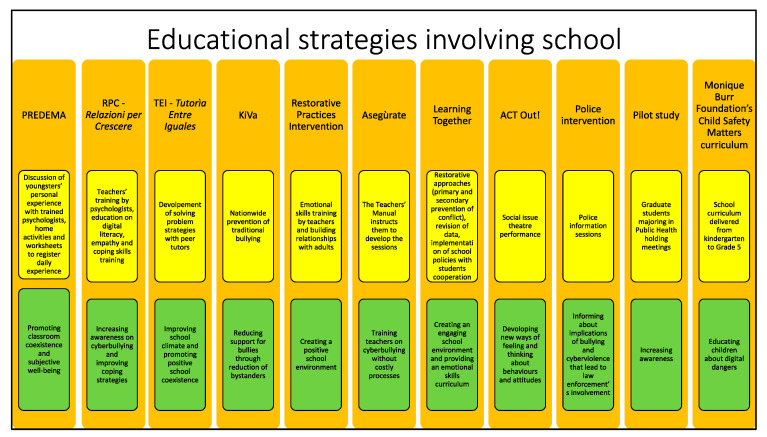
This figure summarizes descriptions and objectives of the educational strategies involving school that will be presented below. The name of the strategy is reported in orange boxes. Yellow boxes contain different strategies’ descriptions, while objectives are stated in the green boxes.

The majority of studies included in this category focus attention on educational activities carried out in school settings during schooltime hours (Figure 2).

In 2018, Schoeps et al. [20] studied an emotional education intervention in the Spanish school setting: PREDEMA. This program was designed with the aim of promoting classroom coexistence (and thus reducing cyberbullying) and subjective well-being. A trained psychologist held the sessions that all started with a personal experience discussed in order to draw a lesson from it and use it for future situations. Students were given home activities and a worksheet to register their daily experience. Results collected immediately after the intervention showed that cyberaggression and cybervictimization had reduced significantly in the experimental group compared to control group, and this effect was still effective at 6 months after the end of the program.

In 2019, Guarini et al. [21] explored the effects of a short intervention developed in Italy (RPC, “*Relazioni per crescere*”—Relationships to Grow) on students’ increased awareness of cyberbullying phenomenon and coping strategies. This program was entirely delivered through teachers previously trained by expert psychologists. In-class activities focused on digital literacy, education on cyberbullying, empathy training, and coping skills improvement. Teachers actively involved students through brainstorming, teamwork, role-playing, and production of posters, slogans, and pictures. This intervention was performed for a period of two months. In the questionnaires administered after that, the number of students acknowledging the different roles in cyberbullying increased, and mentioning keywords related to victimization doubled compared to what had been detected before the intervention. Similar effects were found for coping strategies, whereas a non-significant reduction of cyberbullying or cybervictimization emerged.

In 2019, Ferrer-Cascales et al. [22] examined the effectiveness of Spanish TEI (“*Tutoría Entre Iguales*”—Peer Tutoring) Program focused on the ecological model of peer tutoring. The goal of the program is promoting the improvement of school climate and the promotion of positive school coexistence through developing problem-solving strategies. To do so, given the main role played by peer tutors, teachers, as coordinators, and parents as well are involved in the implementation of the program. Results proved the program was successful in the reduction of fighting, bullying, and cyberbullying victimization and on school climate improvement.

In 2019, Tiiri et al. [23] explored the implication of the introduction of a nationwide anti-bullying program (KiVa) in Finnish schools in 2009. This whole-school intervention considers bullying a group phenomenon and works on reducing bystanders and, in doing so, reducing support for bullies and their motivation to bully. This program focuses mainly on preventing traditional bullying. Results, however, showed a reduction of bullying victimization (especially for boys) and a reduction of frequent cyberbullying victimization only for boys. On the other hand, the sense of security at school increased significantly. In the discussion, the authors highlighted that, although KiVa promotes a strong bystander approach, only 31% of the adolescents reported that other students always or usually tried to stop bullying. They suggest, as an interpretation of the reduction of victimization, a change in the culture since Finland had frequent media coverage on the theme. KiVa targets classroom norms and behaviours in groups, and they differ from those of online social networks.

In 2019, in the USA, Acosta et al. [24] studied the impact of the Restorative Practices Intervention, hypothesizing that a positive school environment, which can be created through a training of emotional skills and by developing relationships with adults, can lead adolescents to experience or perpetrate fewer incidents of bullying. Results showed that Restorative Practices Interventions did not affect cyberbullying but reported the best intervention experiences thanks to their teachers’ actions, reporting less cybervictimization. Hence, even if the intervention itself did not prove effective, a positive effect of a good school environment on cyberbullying indirectly emerged, suggesting that interventions that target improving educational environments are to be considered.

In 2019, Del Rey et al. [25] presented the Asegùrate Program’s impact on cyberbullying. This intervention, developed in Spain, targets teachers, but it overcomes other programs’ need for teachers to take part in costly training processes. In fact, the key component of the Asegùrate Program is The Teachers’ Manual, which instructs on how to conduct the eight sessions planned. Along with the manual, worksheets, and instructions for presenting the program in the classroom, a guide to work together with children’s families and audio–visual materials to be used with pupils were provided. Schools were divided in two groups, of which one carried out the intervention, and the other did not. Both the roles of cybervictim and aggressor were notably lowered in the experimental group after the intervention compared with the control group.

In 2020, in England, Bonell et al. [26] implemented the knowledge of the Learning Together Program by studying its long-term effects (at 24 and 36 months after the intervention). The target of this intervention was creating a more engaging school environment using “restorative approaches” to address conflict, involving students in the revision of data on student experience, and using those to implement school policies and providing an emotional skills curriculum. “Restorative practices” include primary prevention of conflict (through activities aimed at bringing students together and encouraging them to build new relationships) and secondary prevention to resolve incidences once they occur, holding conferences to discuss them together. The present analysis explored the effect on other outcomes, and it found that intervention schools showed lower rates of cyberbullying victimization than control schools at 24 months, whereas at 36 months, they showed reduced rates of cyberbullying perpetration but not victimization. Authors explained this inconsistency of results across time points as a result of chance.

In 2021, Agley et al. [27] explored the effectiveness of ACT Out!, a social issue theatre tested in Indiana, whose scenarios are specifically designed to meet the audience’s needs, although its performances, which were attended during school hours, are improvisational and interactive. The principle upon which this intervention is founded is catharsis, through which a dramatic performance can convey new ways of perception about a situation, in this case, new ways of feeling and thinking about behaviours and attitudes. Although results did not show a superiority of 1 h ACT Out! intervention compared to the standard treatment offered to the control group, evidence of a small reduction of cyberbullying victimization was found, suggesting a promising application of this new type of intervention in view of its particularly short duration.

In 2021, Zafra et al. [28] examined the impact of police information sessions on bullying and cyberviolence held during school time in Spain. Questionnaires self-administered 15 to 30 days after the meeting showed the low impact of this intervention in preventing and clarifying bullying since low perception of bullying involvement and high percentage of inadequate reaction to witnessing bullying episodes emerged.

In 2022, Peng et al. [29] analysed a new intervention and conducted a pilot study in China through some classroom sessions held by graduate students majoring in Public Health, in which various activities were conducted: an introduction on the topic (definition and consequences of bullying), distribution of bullying educational leaflets, playing videos on the consequences of bullying, and a class meeting on strategies to adopt to deal with or prevent bullying. Results showed an increase in awareness of bullying in the experimental group compared to the control group, and cybervictimization decreased significantly in the experimental group compared to the control group. However, there was no significant reduction in cyberbullying.

In 2022, Bright et al. [30], starting from a randomized control trial (RCT) lasting two years, aimed to evaluate the knowledge acquisition of children receiving the Monique Burr Foundation’s Child Safety Matters curriculum in Florida schools (across eight countries), which consists of a program conceived to educate children, from kindergarten to grade 5, about bullying, cyberbullying, four types of abuse (physical, sexual, emotional, and neglect), and digital dangers. Findings illustrate that, on one hand, children who received the curriculum increased their knowledge about potentially risky situations, and on the other hand, children in the control schools did not have similar gains. Therefore, this program is a promising strategy to address children’s vulnerability to cyberbullying and its consequences.

#### 3.1.2. Educational Strategies Involving Families

**Figure 3:**
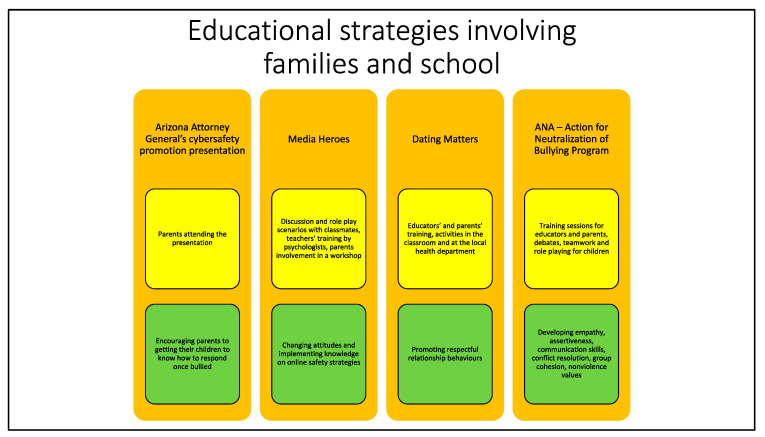
This figure summarizes descriptions and objectives of the educational strategies involving families and school that will be presented below. The name of the strategy is reported in orange boxes. Yellow boxes contain different strategies’ descriptions, while objectives are stated in the green boxes.

Studies involving families describe strategies aimed at sensitizing their members to gain greater control of the online activity of minors and at educating them to recognize warning signs in the behaviour of their children and to address eventual problems with them (Figure 3).

In 2017, Roberto et al. [31] reported the effects of the Arizona Attorney General’s cybersafety promotion presentation for middle-school students’ parents. The focus of this intervention is encouraging parents in helping their children to know how to respond once they have been bullied. The fundamental points to talk about are saving evidence, not retaliating, and telling a trusted adult or authority figure. Parents who viewed the presentation demonstrated that they would perceive their children as more susceptible to cyberbullying and would have greater intentions to talk about the three points mentioned above with their children than those in the control group.

#### 3.1.3. Educational Strategies Involving Both Schools and Families

Here, we illustrate strategies that involve both schools and families (Figure 3).

In 2019, in Germany, Zagorscak et al. [32] developed and examined the Media Heroes intervention program in two versions (a long and a short one), which aimed at changing attitudes and normative beliefs through the transfer of knowledge on cyberbullying and online safety strategies and through discussions and role play with classmates on these themes. Teachers were also involved in this program, as they attended training sessions led by psychologists and were given a manual. Students in the long intervention group involved parents, too, by preparing a workshop for them in which they distributed pamphlets, gave talks, or staged role plays. Results showed how the long intervention group experienced a larger reduction in cyberbullying behaviours, whereas students that attended the short version of the intervention did not differ significantly from the control group (that did not attend any kind of intervention). The long-version intervention was also more successful in reducing somatic symptoms than the short version. According to the authors, these findings suggest a dose–response relationship of this intervention.

In 2021, Vivolo-Kantor et al. [33] explored the effects of the Dating Matters Program compared to Safe Dates, a dating-violence-prevention program that was already proven useful in reducing physical and sexual dating violence victimization and perpetration. Both programs were developed in the USA. Dating Matters involves multiple components targeting individual, relationship, and community levels of the social ecology. In order to do that, it includes classroom-delivered programs, training for parents and educators, youth-communication programs, and activities at the local health department. The ultimate goal is the promotion of respectful relationship behaviours by teaching, for example, skills for emotional regulation, emotional literacy, or the ability to identify, understand, and respond to feelings in a healthy way. Results showed that Dating Matters reduced the relative risk of bullying perpetration (for both males and females) and of cyberbullying perpetration and victimization (only among females) scores more than Safe Dates.

In 2021, in Spain, Martìnez-Martìnez et al. [34] published about the Action for Neutralization of Bullying Program (ANA), which promotes the development of empathy, assertiveness, communication skills, conflict resolution, group cohesion, and coexistence and nonviolence values. Training sessions were organized for both educational community and parents before the start of the children’s program. Children’s sessions had the aim of raising awareness in the observers through activities such as debate, teamwork, and role playing. Results showed that up to 3 months after the intervention, some bullying behaviours decreased as well as bullying victimization, but effects on cyberbullying were not statistically significant.

### 3.2. Digital Strategies

**Figure 4:**
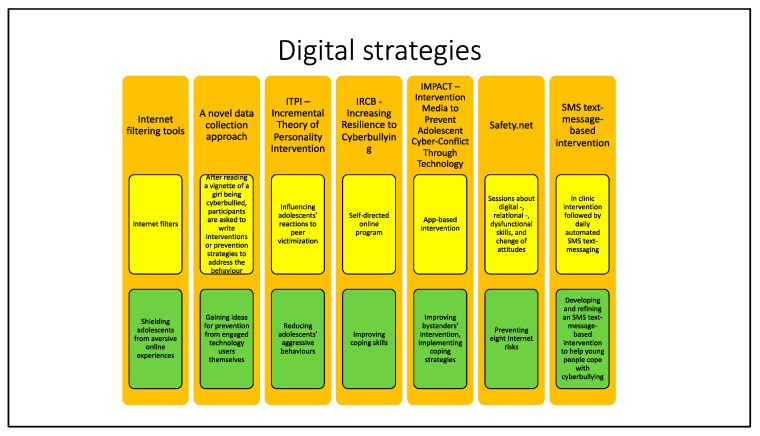
This figure summarizes descriptions and objectives of the digital strategies that will be presented below. The name of the strategy is reported in orange boxes. Yellow boxes contain different strategies’ descriptions, while objectives are stated in the green boxes.

In this section, we clustered some technology-based interventions that have as their objective to set programs to reduce and prevent cyberbullying through the usage of digital instruments and social media (Figure 4).

In 2017, Przybylski and Nash [35] reported upon the effectiveness of Internet-filtering tools designed to shield British adolescents from aversive online experiences. Contrary to the authors’ hypotheses, policy, and industry advice regarding the assumed benefits of filtering, they found convincing evidence that Internet filters were not effective at shielding early adolescents from aversive online experiences. Given this finding, they proposed that evidence derived from a randomized controlled trial and registered research designs are needed to determine how far Internet-filtering technology supports or thwarts young people online. Only then will parents and policymakers be able to make an informed decision as to whether their widespread use justifies their costs based on sound scientific evidence.

In 2018, Selkie et al. [36] furthered the literature by using a novel data collection approach to determine perspectives on electronic harassment intervention and prevention from a targeted group of highly engaged 14/18-year-old adolescent technology users in the USA. This study employed an open-ended qualitative survey format in which, after completing demographic questions, participants read a vignette about a girl being cyberbullied. After that, participants were asked to write interventions and/or prevention strategies to address the electronic harassment behaviour. Results have shown both that adolescents and researchers have different definitions of cyberbullying and that adolescent technology users see cyberbullying as a multi-layered problem with opportunities for intervention in several ecological domains, such as the institutional level, public campaigns, the community level, and state and national harassment policies.

In 2019 Calvete et al. [37] examined, as a part of a project conducted in Bizkaia (Basque Country, Spain) among 10 schools, how the ITPI (Incremental Theory of Personality Intervention), whose effectiveness was found to be moderated by age (12- to 17-year-old students), reduces the reciprocity between victimization and perpetration of cyberbullying. The intervention, divided into three parts and consisting of a double-blind randomized control trial with two parallel groups, reduced the intensity of the association between victimization, which was considered a strong predictor of perpetration of cyberbullying, and perpetration, which was not a predictor of victimization. Results demonstrate that influencing adolescents’ reactions to peer aggression victimization is one of the mechanisms that could explain the beneficial effects of the ITPI in reducing aggressive behaviour in adolescents.

In 2020, in Australia, Chillemi et al. [38] developed a pilot study to test the effectiveness of an online, self-guided cognitive-behavioural therapy-based psychoeducational program for coping with a potential experience of cyberbullying. The IRCB program consists of a classroom-based, self-directed online program structured to include psychoeducation about useful coping skills, guiding examples to reinforce prior learning, open-ended questions to encourage adolescents to think about how the skills may benefit them personally, and vignettes to illustrate how to combine each of the coping skills. The main aims of the increasing resilience to cyberbullying (IRCB) program was to increase adolescents’ likelihood of employing coping skills that may be helpful for a victim of cyberbullying and to increase confidence in their ability to cope and/or help a friend cope with an experience of cyberbullying. Findings have shown, on one hand, significant increases in adolescents’ likelihood of using the coping skills of self-compassion and challenging unhelpful thinking to cope with an experience of cyberbullying and, on the other, significant improvements in adolescents’ help-seeking attitudes and behavioural intentions to engage with counselling services in the event of being a victim of cyberbullying. Despite that, there was no evidence to suggest that the IRCB program significantly increased adolescents’ confidence in their ability to cope and/or help a friend cope with an experience of cyberbullying. Considering all of the above, the authors suggested that an online intervention has the potential to provide adolescents with a free and easily accessible intervention that helps ameliorate the effect of cyberbullying by promoting effective coping skills.

In 2021, Kutok et al. [39] studied IMPACT (Intervention Media to Prevent Adolescent Cyber-Conflict Through Technology), which consists of a brief and remote app-based intervention developed in the USA to reduce the negative effects of cyberbullying and improve bystanders’ intervention. In particular, between 30 January 2020 and 3 May 2020, a national sample of 80 adolescents with a history of past-year cybervictimization was recruited by using some targeted Instagram advertisements for a randomized control trial of IMPACT. Even though this study was not powered for efficacy, results suggest that remote recruitment and enrolment in app-based intervention is highly acceptable and feasible and may be effective in improving well-being, increasing bystanders’ intervention, and growing coping strategies among cybervictims, which can be easily reached through web-based recruitment models.

In 2021, Ortega-Barón et al. [40] explored the effectiveness of the Safety.net program, which was implemented in Spain to prevent eight Internet risks, both relational and dysfunctional, among which there is also cyberbullying, in 11–14-year-old adolescents. The program comprises 16 one-hour sessions to address each risk and divided into four modules: digital skills, relational skills, dysfunctional skills, and change of attitudes and cognitions. In every session, the previous content is recalled so that the program has a networked instructional design in order to maximize the connections between risks and their prevention, generating significant changes between pre- and post-implementation. Results showed that this is an effective program in reducing and preventing cyberbullying even in a small number of sessions.

In 2021, Ranney et al. [41] studied, starting from agile qualitative methods, a mixed-modality intervention initiated within the context of usual paediatric care for adolescents in the USA with a history of cyberharassment and cyberbullying victimization. In this research, two groups of adolescents recruited from an urban primary care clinic participated in three consecutive iterations of the program, which consisted of a brief in-clinic intervention followed by 8 weeks of daily, automated SMS text-messaging. The main objective was to develop, refine, and pilot an SMS text-message-based intervention to help young people to recognize, cope with, and prevent cyberbullying. At the end, results showed that both participants’ satisfaction and participants’ engagement improved considerably with each interaction, so this study shows the value of structured participant feedback gathered in an agile intervention-refinement methodology for the development of a technology-based intervention targeting adolescents.

### 3.3. Hybrid Strategies

**Figure 5:**
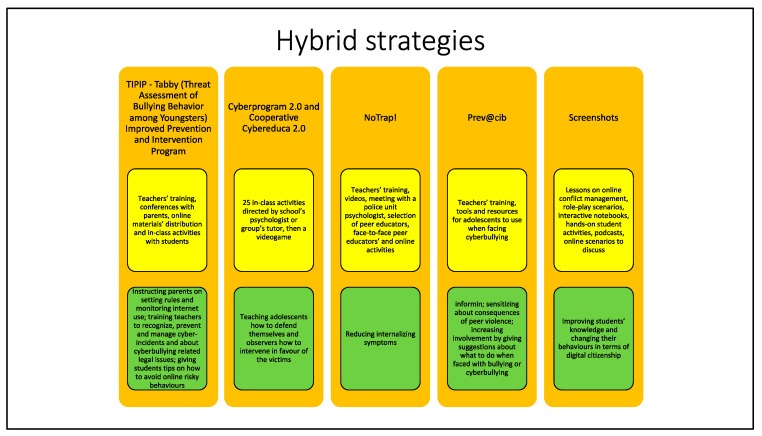
This figure summarizes descriptions and objectives of the hybrid strategies that will be presented below. The name of the strategy is reported in orange boxes. Yellow boxes contain different strategies’ descriptions, while objectives are stated in the green boxes.

In this section, we grouped interventions that use a more comprehensive approach, mixing tools already in use in educational and online strategies (Figure 5).

In 2018, Sorrentino et al. [42] published about The Tabby (Threat Assessment of Bullying Behavior among Youngsters) Improved Prevention and Intervention Program (TIPIP). This intervention, developed in Italy, extends on multiple levels. In fact, on one side, it includes teachers’ training, school conferences with parents, online materials’ distribution, and in-class activities with students, which aim at informing, defining, and sensitizing the parts in the phenomenon. On the other side, there are aspects concerning the specific target of the activity. For example, parents are instructed on how to protect their children by setting rules on Internet use and by monitoring their online activities; teachers are trained to recognize, prevent, and manage cyber-incidents and are informed about legal issues connected with cyberbullying; and students are given tips on how to avoid online risky behaviours and involvement in cyberbullying. Both cyberbullying and cybervictimization decreased in the experimental group compared to the control after the intervention. Analysing results and distinguishing for sex, it emerged that the decrease in both cyberbullying and cybervictimzation was statistically significative for boys but not for girls.

In 2018, Garaigordobil et al. [43] investigated an intervention proposal validated experimentally in Spain and consisting of a program (Cyberprogram 2.0) and a videogame (Cooperative Cybereduca 2.0), which aims to prevent and reduce cyberbullying during adolescence. This prevention instrument is composed by, in the first part, 25 activities carried out weekly in the classroom during the school year with sessions of an hour directed by the school psychologist and/or the groups’ tutor in order to fulfil four main objectives that are directed to teach adolescents how to defend themselves and to teach to observers how to intervene in favour of the victims and in general to provide them with strategies to prevent and deal with cyberbullying in all its forms. On the other hand, in the second part, there is a videogame, namely a trivial pursuit game, organized around a fantasy story, with questions and answers related to bullying and cyberbullying, played online and free of charge. Results have shown that there was a decrease in cyberbullying behaviours and in different types of school violence and an increase of positive social behaviours, self-esteem, cooperative conflict-resolution strategies, and the capacity for empathy.

In 2019, Palladino et al. [44] improved the knowledge of the efficacy of the Italian NoTrap! Program studying the effects of internalizing symptoms. This program consists of different parts: teacher training, presentation of the program to the participating classes using videos and a meeting with a special police unit psychologist, selection of peer educators, face-to-face peer educators’ activities in their own classes, and online activities. Not only was a reduction of internalizing symptoms on the experimental group found, but they also linked it to the reduction of cybervictimization itself. In fact, the decrease in victimization and in cybervictimization completely accounted for the effect on internalizing symptoms.

In 2019, Ortega-Baron et al. [45] studied Prev@cib, a Spanish program divided into three modules: the first aims at informing about the problem, its risk factors, and strategies to avoid becoming involved in potential cyberbullying problems. The second aims at sensitizing about the consequences of peer violence both at school and in using technologies. The third one aims at increasing involvement by giving suggestions about what to do when faced with bullying or cyberbullying. Results showed a decrease in both cyberbullying and cybervictimization in the group exposed to the intervention, whereas they remained stable and increased, respectively, in the control group. However, these are only short-term effects since post-test questionnaires were administered soon after the end of the program.

In 2021, Bickham et al. [46] studied the effects of Screenshots, a program developed in the USA that aims at improving students’ knowledge and at changing their behaviours in terms of digital citizenship. Screenshots consists of lessons about managing online conflict and practicing empathy through different activities such as role-play scenarios, interactive notebooks, hands-on student activities, podcasts, and online scenarios to discuss. After the intervention, questions answered about digital citizenship showed a change in students’ beliefs towards being less supportive of unkind online behaviours and more supportive of privacy and safety. About conflict resolution, males answered showing less inclination towards the use of verbal aggression, whereas results for females went in the opposite direction.

### 3.4. Other Strategies

**Figure 6:**
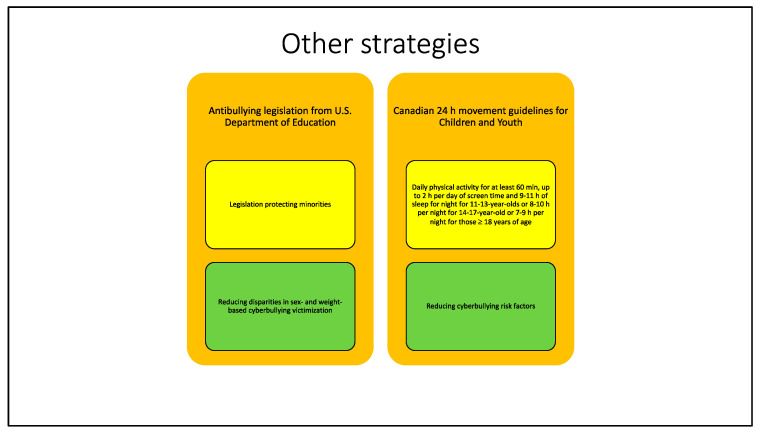
This figure summarizes descriptions and objectives of the other strategies that will be presented below. The name of the strategy is reported in orange boxes. Yellow boxes contain different strategies’ descriptions, while objectives are stated in the green boxes.

Recent research and studies suggest that antibullying laws are a potential instrument in reducing risk of cyberbullying victimization among youth and adolescents (Figure 6).

In 2017, Hatzenbueheler et al. [47] tried to evaluate the effectiveness of anti-bullying legislation in reducing disparities in sex- and weight-based cyberbullying victimization. This investigation was conducted starting from data on anti-bullying legislation taken from the U.S. Department of Education, which commissioned a systematic review of 16 key components of state laws in 2011. States were categorized based on whether their legislation enumerated protected groups and if so, which groups were enumerated. These policy variables from 28 states were linked to individual-level data on bullying and cyberbullying victimization from students in 9th through 12th grades participating in the 2011 Youth Risk Behavior Surveillance System study. At the end, findings demonstrated that these kinds of policies were not associated with lower weight-based disparities in cyberbullying victimization among youth. Despite that, policies with high compliance with the Department of Education enumeration guidelines were associated with a lower sex-based cyberbullying victimization.

Another way of addressing cyberbullying is through prevention, acting on the reduction of risk factors (Figure 6).

In 2020, Sampasa-Kanyinga et al. [48] investigated the impact of the “Canadian 24 h movement guidelines for Children and Youth” on bullying and cyberbullying involvement among adolescents. Daily physical activity for at least 60 min, up to 2 h per day of screen time, and 9–11 h of sleep for night for 11–13-year-olds or 8–10 h per night for 14–17-year-old or 7–9 h per night for those ≥18 years of age were considered variables meeting the Canadian recommendation. Results showed that meeting all three recommendations had the strongest association with bullying outcomes; in particular, meeting the screen time recommendation was associated with lower odds of being a victim or a bully, meeting screen- and sleep-time with lower odds of being a bully, and meeting all three recommendations with lower odds of being a victim or a bully-victim compared to those who did not meet any recommendation. With regards to cyberbullying, meeting the screen time recommendation only or meeting all three of them was associated with lower odds of being a cyber-victim. Meeting the screen time recommendation only was associated with lower odds of being both a school bullying and a cyberbullying victim and both a school and a cyberbully; meeting screen and sleep time recommendations was associated with lower odds of being both a school and a cyberbully, while meeting all three recommendations with lower odds of being a school bullying or a cyber victim and both school and cyberbully.

## 4. Discussion

Nowadays, digital violence and aggression is a highly prevalent problem among adolescents, especially for the fact that, in many cases, victimization and perpetration tend to overlap since several youngsters, motivated by the desire to take revenge, react with violence when they are victims of aggression [37]. Since cyberbullying may occur anywhere and anytime, being independent of a specific context such as school [49], it is necessary that strategies to combat it adopt an integrated and multilevel approach including schools, families, and political institutions in order to create a synergic intervention. In this sense, it is important to promote a positive use of technologies among children and adolescents, starting from families and schools, which are the first places where youngsters’ education begins. In particular, schools should implement their curricula by including specific anti-cyberbullying programs in order to increase awareness in its students [50].

**Figure 7:**
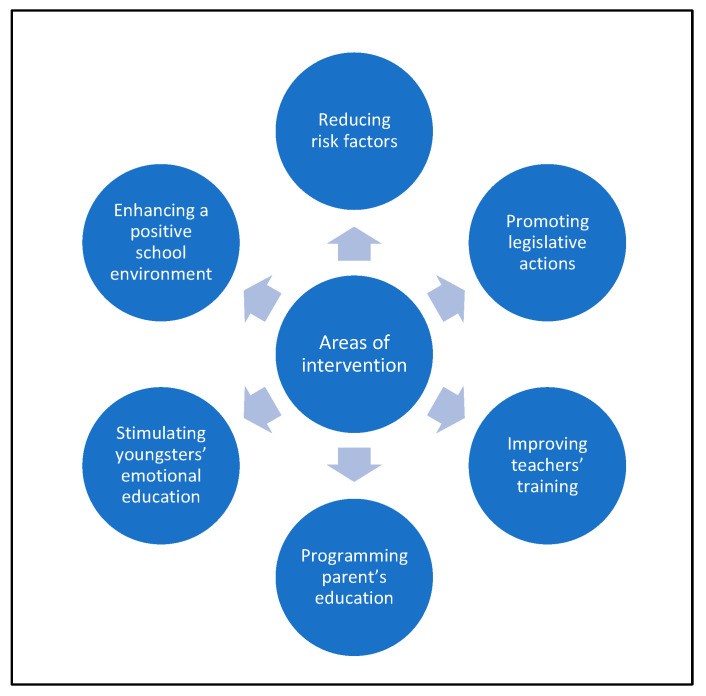
This figure represents the areas of interventions studied by articles included in the results of this review.

A schematic representation of areas of interventions explored and tested by articles included in our review is reported in Figure 7.

The majority of the interventions presented in this review aim at increasing critical awareness of digital knowledge and not only giving young people prescriptive and juridical indications, since the only intervention of the latter type included in this review and consisting of police information sessions did not prove to be much effective [28]. The majority of the interventions are intended to train children and adolescents to be active and responsible citizens of the digital world, knowing their rights and duties and the importance of respecting rules, in order to promote online positive behaviours. To do so, many of them consist of a first step of sensitization and a second step of minors’ active involvement through activities such as teamwork, role-play scenarios, production of posters or leaflets, etc., as in RPC (“*Relazioni per Crescere*”—Relationships to Grow) [21], in the pilot study by Peng [29], and in Screenshots [46]. With the same objectives in mind, other interventions point to stimulating youngsters’ emotional education in a less conventional way by interacting in a theatre performance on the theme (as in ACT Out! [27]) or by developing apps, videogames, or automated SMS text-messaging to deliver the intervention remotely (as in IMPACT [39], in Cooperative Cybereduca 2.0 [43], and in the study by Ranney [41], respectively).

What has emerged from our results is that school is the most targeted setting for cyberbullying interventions to take place. If, on one hand, school is the most used place to stimulate minors’ emotional education, on the other hand, it is itself target of some interventions that aim at enhancing a positive school environment, such as PREDEMA [20] and TEI (“*Tutorìa Entre Iguales*”—Peer Tutoring) [45]. Other interventions mix these aspects, hypothesizing that the key to creating a positive environment is emotional education itself, as in the Restorative Practices Intervention [24] or in the Learning Together Program [26].

Although some of the interventions included in our results explore the effects of teachers’ and parents’ training, these represent only a minority. Given the key role of these figures in children’s and adolescents’ development and the promising results obtained by the Arizona Attorney General’s cybersafety promotion presentation [31], the Media Heroes Program [32], the Dating Matters Program [33], and the Action for Neutralization of Bullying Program [34], it would be appropriate for further programming to include teachers’ and parents’ training and educational interventions in the near future. However, there are other figures and settings frequently visited by youngsters that may be included in future programs, for example, those related to extracurricular activities.

Even if there are many studies in the literature analysing the correlation between different variables and bullying or cyberbullying involvement, few of them analyse the modification of such risk factors as a specific type of intervention targeting cyberbullying. We also included in this systematic review two studies belonging to this latter group: in particular, the first one explores Internet-filtering tools [35] as instrument to shield adolescents from potentially risky online situations, while the second one evaluates the efficacy of recommendations regarding daily physical activity or screen- and sleep time [48]. For these kinds of studies, it should be noted that it remains difficult to monitor the real impact of a modification of cyberbullying risk factors on cyberbullying involvement.

As said above, even if schools, as the main educational setting, cannot ignore the vastity of the phenomenon, a comprehensive approach including media-campaigns, school programs, teachers’ and parents’ involvement, legislative actions, and screening and interventions by paediatricians and healthcare professionals has been suggested as more appropriate [51]. The analysis of the results obtained in our review has underlined the importance of such interventions: for example, the NoTrap! Program [44] or The Tabby Improved Prevention and Intervention Program [42] include training for parents, teachers, and other figures and intervention in the following areas: communication skills, empathy, and increase in self-esteem.

Another aspect that emerged from our results is how programs targeting bullying alone or both bullying and cyberbullying are often effective also on both bullying and cyberbullying outcomes. This supports the theory that the cyber context has offered a new dimension to the already-known offensive roles of bullying [52]. In this sense, interventions programmed for the contrast of bullying, such as KiVa [23], developed mainly as having traditional bullying as a target, have proven effective on cyberbullying predictors as well.

This review offers some insights that need to be further explored by future studies: first, as mentioned above, the need of a better knowledge of response-predictive variables in relation to cyberbullying interventions; second, the majority of the studies included here explore the short-term effects of the programs, whereas findings from the study of Tiiri et al. on KiVa show that the longer students attended the program, the better the outcome, suggesting a positive dose effect of this intervention [23]. The same suggestion was made by Zagorscak et al., seeing that the longest version of the Media Heroes intervention had the best outcome on cyberbullying behaviours compared to the short version, offering evidence of the positive dose effect for cyberbullying programs as well [32].

Considering the importance of digital education in cyberbullying prevention and in the reduction of online aggressive behaviours, future interventions could program and implement some educational mobile apps that are available everywhere and at any time on different devices, in which young people could find useful information about the main Internet risks and their prevention. Moreover, such apps could serve as platforms through which the adolescents could send help requests when they fall into an episode of cyberbullying. In this way, it may also be possible to create some virtual communities where youngsters can find some psychological support and where they can be encouraged to report those negative experiences.

This review’s findings should be interpreted in light of our work’s limitations. We conducted a literature search applying an English-language filter; therefore, significant findings in other languages might have been overlooked. Similarly, we used a single database (*PubMed*), and we might have omitted a relevant search term and thus consequently not retrieved other relevant results. In addition to this, our review’s transversal nature and characteristics of included studies preclude from drawing causal relationships or making a quantitative comparison of the studies’ results.

## 5. Conclusions

This review offers an insight on the programs already tested for efficacy in preventing and contrasting cyberbullying, showing on one hand an increasing attention to the phenomenon and on the other hand how difficult it is to intervene in such complex dynamics.

Studies in this review generally show a positive outcome on cyberbullying indicators.

However, not all the strategies exposed among our results have, in fact, proven to be effective in reducing indicators of cyberbullying, and for the majority of those that did prove to be effective, long-term effects need to be further assessed through future studies.

Children’s and adolescents’ educational interventions are indeed the most studied, but an integrated approach might be a useful tool. In fact, comprehensive approaches involving mental health professionals’, educators’, and digital experts’ participation in programs for children and adolescents’ personal growth, although promising strategies at present, are to be progressively implemented and validated in the effort of updating educational messages to the modern world’s needs.

Future research should go further and expand both the sample size and the time after the intervention before measuring its effects. It could also be interesting to explore already-known settings for interventions, performing more studies on families and educators, as well as developing new interventions taking place at different settings, for example, at extracurricular activities.

## Data Availability

No publicly archived datasets were created. Data are available upon request to the corresponding Authors.
